# Inferring the conservative causal core of gene regulatory networks

**DOI:** 10.1186/1752-0509-4-132

**Published:** 2010-09-28

**Authors:** Gökmen Altay, Frank Emmert-Streib

**Affiliations:** 1 Computational Biology and Machine Learning, Center for Cancer Research and Cell Biology, School of Medicine, Dentistry and Biomedical Sciences, Queen's University Belfast, 97 Lisburn Road, Belfast, BT9 7BL, UK

## Abstract

**Background:**

Inferring gene regulatory networks from large-scale expression data is an important problem that received much attention in recent years. These networks have the potential to gain insights into causal molecular interactions of biological processes. Hence, from a methodological point of view, reliable estimation methods based on observational data are needed to approach this problem practically.

**Results:**

In this paper, we introduce a novel gene regulatory network inference (GRNI) algorithm, called C3NET. We compare C3NET with four well known methods, ARACNE, CLR, MRNET and RN, conducting in-depth numerical ensemble simulations and demonstrate also for biological expression data from *E. coli *that C3NET performs consistently better than the best known GRNI methods in the literature. In addition, it has also a low computational complexity. Since C3NET is based on estimates of mutual information values in conjunction with a maximization step, our numerical investigations demonstrate that our inference algorithm exploits causal structural information in the data efficiently.

**Conclusions:**

For systems biology to succeed in the long run, it is of crucial importance to establish methods that extract large-scale gene networks from high-throughput data that reflect the underlying causal interactions among genes or gene products. Our method can contribute to this endeavor by demonstrating that an inference algorithm with a neat design permits not only a more intuitive and possibly biological interpretation of its working mechanism but can also result in superior results.

## Background

The inference of large-scale causal gene regulatory interactions is important because it can contribute to a better understanding of all aspects of normal cell physiology, development and pathogenesis [1-3]. The orchestral interaction among genes and gene products manifests in gene networks of an organism, e.g., the transcriptional regulatory network, protein network or metabolic network [4-7]. These networks represent blueprints of dynamical processes within cells. Different types of gene networks are distinguished by highlighting different perspectives of these dynamical processes, e.g., the regulation of the transcription of genes or the formation of protein complexes or metabolic reactions [8,9]. For this reason, the inference of gene networks from experimental data has been named as one of the most prominent goals of the post-genomic era and in systems biology. Classical molecular biology approaches *in vivo *or in *vitro *allow an accurate detection of molecular interactions, however, they are laborious and limited in the number of interactions that can be studied [10]. In contrast, due to recent advances in biotechnology, nowadays, the measurement of large-scale expression data, quantifying the concentration of mRNAs, on the genomic level is feasible. The availability of this type of data ushered the development of methods inferring and analyzing gene regulatory networks [11], a specific type of a gene network. The challenge of this problem is that expression data are frequently limited to observational data only and no randomized or interventional data can be generated because of either technological, economical or ethical conditions. Specifically, the major problem in this context, and the main topic of this paper, relates to the inference of causal interactions among genes from observational data. This problem has already received considerable interest and is, aside from biology, of the utmost interest in a series of fields like economics, epidemiology, medicine, sociology and statistics [12-15].

So far several methods have been suggested in the above context, inferring gene regulatory networks [16-19]. The best of these are based on information theory [20,21], estimating mutual information (MI) values and combine these estimates via step-wise procedures [22-25]. One of the first methods introduced was RN (Relevance Network) [22]. This method estimates pairwise mutual information values among all genes and eliminates the edges among genes that have mutual information values that are not statistically significant. Similar to RN is ARACNE (Algorithm for the Reconstruction of Accurate Cellular Networks) [24]. However, in addition to the steps described for RN, ARACNE makes use of the data processing inequality (DPI) [26] to eliminate the least significant edge of a triplet of genes, which corresponds to the lowest mutual information value thereof. This gives a more conservative estimate of the inferred network because ARACNE can contain at most as many interactions as inferred by RN.

This is due to the application of the DPI, which can only eliminate, but not add edges to the network. Another method similar to RN is CLR (Context Likelihood of Relatedness) [23] which employs a background sensitive estimator for the connection among genes by converting mutual information estimates into z-score like values. Finally, MRNET (maximum relevance/minimum redundancy Network) [25] has been introduced employing the maximum relevance/minimum redundancy (MRMR) feature selection method [27,28].

The major purpose of this paper is to introduce a new inference method. The motivation to suggest a new method is at least three fold. First, the capabilities of previously introduced methods are only partially investigated. This results from the fact that an inference method needs to be studied in combination with data because its performance depends crucially on the characteristics of the data. However, there is neither a general agreement how to simulate data in a way that they would capture all relevant aspects of real expression data, nor we are in possession of a true regulatory network of a reasonable size representing all causal interactions actually involved in a certain physiological process. Further, we do not have access to microarray data of arbitrary large sample sizes due to economic and experimental limitations. Hence, the principle approaches currently pursued for the statistical investigation of an inference algorithm represent a compromise acknowledging the above circumstances. In order to obtain the most thorough analysis of an inference algorithm we analyze our method with an ensemble of simulated data and with biological expression data from microarray experiments of an organism for which, at least to a certain degree, information about the underlying regulatory network is known. Second, the inference algorithms described above have the tendency of becoming more and more complex. Keeping in mind that previous results may be flawed due to the serious difficulty of obtaining a balanced statistical analysis, we step in the other direction aiming for an inference algorithm that is simpler than most other methods. This may not only allow for a better understanding of the proposed method but also reveal something about the underlying biology itself. Third, all previous methods aim, at least theoretically, to infer the entire regulatory network for a given data set. However, practically, no method can guarantee to achieve this for a given data set, not even for simulated data when a very large number of samples is available. One reason for this shortcoming is that observational data may not capture all dynamical interrelations that would allow a reliable estimation. For this reason, we lower the bar from the beginning by not aiming to infer the entire network, instead, our method aims to infer the strongest interactions among covariates only. We call this part of a network its *conservative causal core *or C3.

The basic idea of our method, we call C3NET, consists in the identification of a significant maximum mutual information network, the conservative causal core, in a way that two genes are only connected with each other if their shared significant mutual information value is at least for one of these two genes maximal with respect to all other genes. Since C3NET is an information theory based method, we compare it with ARACNE [24], MRNET [25], RN [22] and CLR [23] for simulated as well as expression data from *E. coli*. With these data, we demonstrate that C3NET gives better results than all other inference methods and in addition has a computational complexity that is among the lowest.

The paper is organized as follows. In the next section we introduce our method, C3NET, and describe its working mechanism. Also, we describe our simulation set-up and the expression data we use. Then we present numerical results comparing our method with ARACNE, MRNET, RN and CLR and application of C3NET to the expression data from *E. coli*. We finish this article with conclusions.

## Methods

In this section we introduce our inference algorithm, C3NET, describe its constituting components and present an example of its working mechanism. In addition, we motivate its introduction and discuss its biological plausibility.

In the first step of C3NET we want to eliminate nonsignificant connections among gene pairs. This can be accomplished by testing the statistical significance of pair-wise mutual information (MI) values employing resampling methods, similarly to previous methods, e.g., RN or ARACNE [22,24]. Mathematically, the mutual information [26] of two random variables *X *and *Y*, taking on values in X and Y, is defined as

(1) $$I(X,\;Y)=\sum_{x\in X}\sum_{y\in Y}p(x,\;y)\log\frac{p(x,y)}{p(x)p(y)}.$$

Practically, the mutual information values need to be estimated from the data by using an appropriate estimator allowing a close approximation of the theoretical value of the population. A discussion of technical details of this issue is provided at the end of the section 'Simulated and expression data'. Starting from a fully connected matrix *C*, with *C_ij _*= 1 for all *i*, *j *∈ *V *and a zero matrix *A*, we test exhaustively all pair-wise mutual information values *I_ij_*, *i*, *j *∈ *V*, and set *C_ij _*= *C_ji _*= 0 if we cannot reject the null hypothesis *H*_0 _: *I_ij _*= 0, for a given significance level *α*. In the second step of C3NET, we first determine the neighborhood *N_s _*for all genes *i *∈ *V*. The neighborhood of gene *i *is defined by *N_s_*(*i*) = {*j *: *C_ij _*= 1 and *j *≠ *i*}. For this purpose we introduced the auxiliary connectivity matrix *C*. From *N_s _*and *I *we determine for each gene the connection to its neighborhood that has the maximum mutual

**Algorithm 1 **Principle steps of our inference algorithm C3NET.

1: *A*: initiate adjacency matrix, *A_ij _*= 0 for all *i*, *j *∈ *V*

2: *C*: initiate connectivity matrix, *C_ij _*= 1 for all *i*, *j *∈ *V*

3: estimate mutual information *I_ij _*for all *i*, *j *∈ *V*

4: **repeat**

5:   Set *C_ij _*= 0 if *I_ij _*= 0 is not statistically significant (hypothesis test)

6: **until **all pairs *i *≠ *j *are tested

7: **for all ***i *∈ *V ***do**

8:   *N_s_*(*i*) = {*j *: *C_ij _*= 1 and *j *≠ *i*}

9:   **if ***N_s_*(*i*) ≠ ∅

**10**:      $j_{c}(i)=\arg\max_{j\in N_{s}(i)\{I_{ij}\}}$

**11**:   **else**

**12**:      *j_c_*(*i*) = ∅

**13**:   **endif**

14: **end for**

15: **for all ***i *∈ *V ***do**

16:   **if ***j_c_*(*i*) = ∅

**17**:      $A_{ij_{c}(i)}=A_{j_{c}(i)i}=1$

**18**:   **endif**

19: **end for**

20: **return **adjacency matrix *A*

information value. This connection is identified by

(2) $$j_{c}(i)=\underset{j\in N_{s}(i)}{\text{argmax}}\{I_{ij}\}.$$

**Figure 1:**
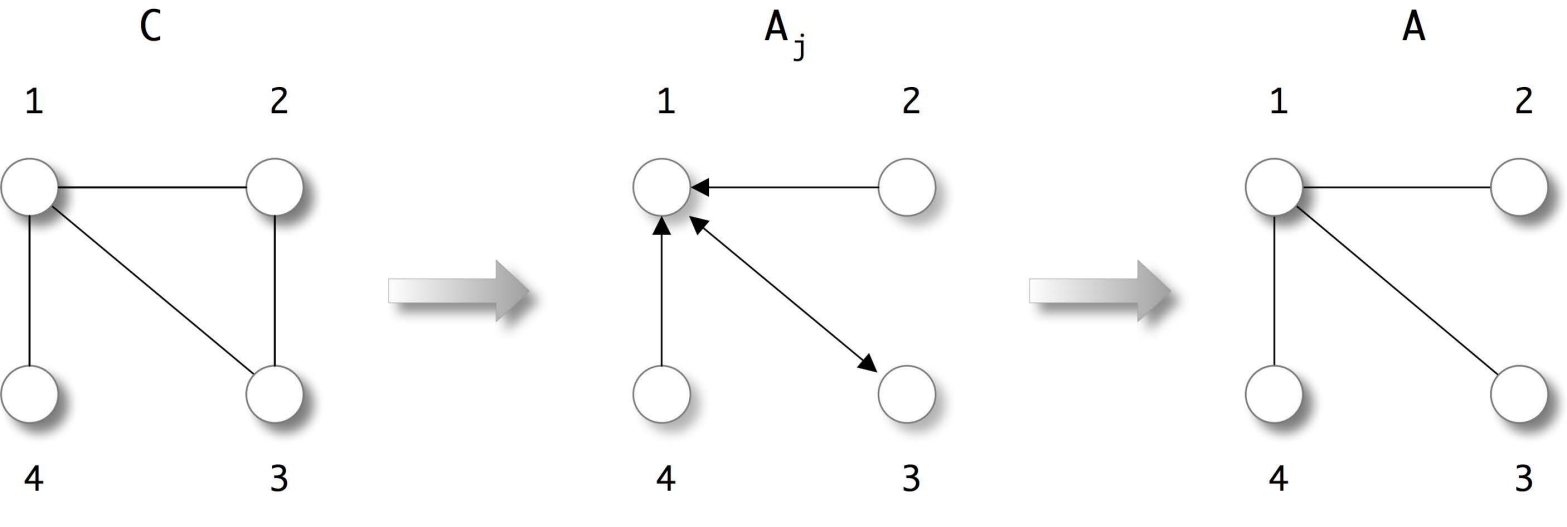
**Visualization of the principle working steps of C3NET and the fact that the final network can have an arbitrary structure**.

In the case *N_s_*(*i*) ≠ ∅ which happens if all mutual information values *I_ij _*for *j *∈ *V *are non-significant, we do not assign an index to *j_c_*(*i*) but the empty set. From this information we construct the adjacency matrix *A *of the estimated undirected network by setting $A_{ij_{c}(i)}=A_{j_{c}(i)i}=1$ if *j*_*c*_(*i*) has been set to a valid index. All other entries of *A *remain zero. The principle steps of our method are summarized in Algorithm 1. The underlying idea that lead us to the introduction of our second step can be motivated as follows. We learned from investigations of various network inference algorithms and their performance analysis employing local network-based measures, instead of global measures like the F-score or AUROC, that the inference of regulatory networks is in general very heterogeneous with respect to different structural regions within the regulatory network. This means, there are substructures in the network, e.g., individual edges, network motifs or subnetworks that exhibit enormous differences in their inferability with respect to an inference algorithm. This observation suggests that the uniform application of an inference algorithm that shows a noticeable bias in its performance induced by structural elements of the network is a suboptimal strategy. Statistically, we found that a reason for these performance variations is given by a strong dependency of the joint probability distributions of expression values on the network structure on which estimates of mutual information values are based on principally. We found similar observations for various inference algorithms (ARACNE, CLR, RN, MRNET) we studied and, hence, it is not a property of a specific inference method, but all current approaches seem to suffer from this limitation. To minimize this problem, based on our findings, we propose a modified *extremal strategy *and hypothesize that the application of a statistical estimator should be minimized to the maximum valued mutual information values, if applied uniformly, in order to maximize the performance of the inference algorithm. Our approach presented above represents the most conservative procedure consistent with our hypothesis, because it allows each gene to add *at most *one connection to another gene in the inferred network. This connection corresponds to the maximum mutual information value between this gene and all its neighbors, which, statistically, has also the lowest p-value. We want to emphasize that despite the fact that one gene can add at most one new edge, finally, a gene can be connected with more than one gene. We demonstrate this with a simple example consisting of four genes. Fig. 1 visualizes our example. Suppose we have the following mutual information values *I *and its corresponding connectivity matrix *C*, as a result of hypotheses tests, given by

(3) $$I=\left(\begin{array}{cccc} 1.0 & 0.7 & 0.9 & 0.8\\ 0.7 & 1.0 & 0.6 & 0.5\\ 0.9 & 0.6 & 1.0 & 0.1\\ 0.8 & 0.5 & 0.1 & 1.0 \end{array}\right)\;\text{,}\;C=\left(\begin{array}{cccc} 1 & 1 & 1 & 1\\ 1 & 1 & 1 & 0\\ 1 & 1 & 1 & 0\\ 1 & 0 & 0 & 1 \end{array}\right).$$

For each of the four genes we determine its connection with neighboring genes with maximum mutual information that is also statistically significant, resulting in *j_c _*= (3, 1, 1, 1). We want to remark that mutual information values that are not statistically significant appear as zero entries in the matrix *C*. From *j_c _*one can determine an auxiliary matrix,

(4) $$A_{j}=\left(\begin{array}{cccc} 0 & 0 & 1 & 0\\ 1 & 0 & 0 & 0\\ 1 & 0 & 0 & 0\\ 1 & 0 & 0 & 0 \end{array}\right),$$

containing exactly the edges added by each node. Since MI does not provide directional information, due to its symmetry in its arguments, the resulting adjacency matrix *A *is a symmetric adjacency matrix.

(5) $$A=\left(\begin{array}{cccc} 0 & 1 & 1 & 1\\ 1 & 0 & 0 & 0\\ 1 & 0 & 0 & 0\\ 1 & 0 & 0 & 0 \end{array}\right).$$

From Fig. 1 one can see that the resulting network represented by adjacency matrix *A *is star-like and gene 1 is connected to 3 other genes. As one can see from our simple example, this results from the conversion of the asymmetric matrix *A_j_*, which was directly obtained from *j_c_*, to a symmetric matrix *A*. Hence, despite the fact that each gene can add at most one connection, different genes *i *can select the same gene *j_c_*(*i*), as in the above example. For this reason, genes can have more than one connection to other genes in the final undirected network.

In addition to the statistical justification sketched above, the working mechanism of C3NET has also a very appealing interpretation from a biological point of view. Genes that are expressed in a cell have to interact with at least one other gene or gene product, because otherwise they could be knocked out without noteworthy effect on the cell's physiology. That means, active genes must have, at least, one connection with other genes in order to contribute to the biological function of the cell. This interaction is targeted by C3NET. On the other hand, if a gene is not expressed in a specific cell type, but the measurements reflect merely noise, the significance test applied in the first step of C3NET prevents the assignment of obviously false positive connections, because the mutual information values are in such a case not statistically significant.

In order to clarify differences between C3NET and other algorithms, we want to discuss some of these. MRNET is based on the *maximum relevance and minimum redundancy *feature selection method [25], which is a significantly different procedure than the one employed by C3NET but also ARACNE, RN or CLR. The method employed by RN corresponds to the first step of C3NET but also of ARACNE, however, both methods employ a second step. In this second step ARACNE utilizes the data processing inequality (DPI) [26] whereas C3NET does not. Instead, we are selecting maximum mutual information values only, among all significant edges, with respect to each gene. Lastly, CLR uses a different estimation method based on z-scores for assessing significance which is different to all other methods.

A characteristic of C3NET that is different to all other methods is that it can infer at most as much edges as genes. The reason for this is that the maximization step allows each gene to add at most one edge to another gene. All other methods are capable of inferring, potentially, more edges than genes. Put differently, this implies that C3NET does not aim at inferring the entire network underlying gene regulation, instead, it aims at its core structure and, hence, it is more conservative than all other methods. The purpose of this paper is to introduce C3NET and to investigate the capabilities of our method by providing a systematic comparison with other inference methods.

### Complexity

The computational complexity of all methods used in this paper, except for C3NET, were discussed in [25]. There the complexity is evaluated assuming that the mutual information matrix is already computed, since all methods are based on it, contributing a complexity of *O*(*n*^2^). In the following, *n *represents the number of genes. The complexity of RN and CLR is *O*(*n*^2^) since only pairwise interactions are evaluated. The complexity of ARACNE is *O*(*n*^3^) because all triplets of genes need to be evaluated for the data processing inequality. The complexity of MRNET is between *O*(*n*^2^) and *O*(*n*^3^) because of the feature selection step, see the discussion in [25]. From the pseudo code of our algorithm in Algorithm 1, one can derive that the computational complexity of C3NET is *O*(*n*^2^) because only matrices of size *n × n *enter our procedure. Hence, the computational complexity of C3NET is also desirably low and among the fastest algorithms. We want to emphasize that all of the algorithms discussed above, have a much lower complexity than Bayesian networks, which for structural learning in general are NP-hard [29,30]. For this reason, in practical applications some sort of heuristic approximation needs to be employed, e.g., [31].

### Simulated and expression data

In order to analyze our proposed inference algorithm by comparing it with the performance of other methods we use simulated as well as expression data from microarray experiments. Due to the fact that the knowledge about biological regulatory networks is still far from being complete, we use simulated data because for these data we know the underlying (true) regulatory network exactly. This allows a detailed and accurate analysis. We complement our simulation study with biological expression data to demonstrate that the assumptions made for our simulations are realistic enough to extrapolate these results to biological data sets.

The error measure we use to assess the performance of an inference algorithm is the F-score, *F *= 2*pr/*(*p *+ *r*). Here precision, *p *= *TP*/(*TP *+ *FP*), and recall, *r *= *TP*/(*TP *+ *FN*), is a function of the number of true positive (TP), false positive (FP) and false negative (FN) edges in an inferred network. In order to analyze the capabilities of an inference algorithm, instead of its employed statistical estimators, we follow [25] to obtain an optimal cut-off value *I*_0 _for the mutual information values by maximizing the F-score. The two biological networks we use in our simulation study represent subnetworks of the transcriptional regulatory network (TRN) of *E. coli *[32,33] and *Yeast *[34]. These subnetworks were randomly sampled with the *neighbor addition *method from these TRNs using SynTReN [35]. Both networks consist of *n *= 100 nodes (genes). With SynTReN [35] we generate synthetic expression data (including noise) mimicking the mRNA concentration in steady-state condition by using non-linear transfer functions based on Michaelis-Menten and Hill enzyme kinetic equations [36-38]. In general, for our simulations, we perform an ensemble approach as in [39,40].

For each network *G *we generate an ensemble of *k *∈ *N *different expression data sets, $X_{ij}^{k}$, each consisting of *j *∈ *p *samples and *i *∈ *n *genes. The data sets differ from each other by the parameters of the kinetic equations used to generate expression values emulating biological variability which is characteristic for biological systems. This results in *N *different F-scores *F_k_*, *k *∈ *N*, for a network *G*. It is important to emphasize that the usage of an ensemble of data sets allows to reveal the characteristics of an inference algorithm relentlessly, because it provides information about the distribution of a performance measure, instead of a single value, for assessing the inference algorithm [39]. On a practical note, in oder to estimate the mutual information values for the synthetic data sets we, first, copula-transform the data, similarly as in [24]. Then we use a parametric Gaussian estimator to estimate MI values, as described in [25] and [41], giving MI value estimates by

(6) $$I(X,\;Y)=(1/2)\log(\frac{\sigma_{X}^{2}\sigma_{Y}^{2}}{\left|C\right|}).$$

Here $\sigma_{X}^{2}$ and $\sigma_{Y}^{2}$ is the variance of *X *respectively *Y *and |*C*| is the determinant of the covariance matrix. Other MI estimators (for instance Miller-Madow, Shrinkage or Schurmann-Grassberger [25,42]) could also be used but since they did not lead to a noticeable difference in the performance we selected the fastest estimator for our simulations. For ARACNE we set the tolerance parameter for the DPI to 0.1, as suggested in [24].

The biological expression data we use in our study is a data set of *E. coli*, we obtained from the supporting information web site of [23]. The data set consists of 524 published *E. coli *Affymetrix microarrays collected under various conditions such as growth phases, varying oxygen concentrations, pH changes, antibiotics, heat shock and numerous genetic perturbations. In order to obtain a reference network that can be used to study the performance an inference algorithm a curated network has been assembled mostly based on the RegulonDB database [43]. This reference regulatory network, $G_{2007}^{EC}$ (the number indicates the year the network was assembled), consists in total of 3091 experimentally confirmed regulatory interactions between 152 transcription factors and 1146 regulated genes. We want to emphasize that the interactions have been limited to these genes, hence, resulting in a none-square 152 × 1146 adjacency matrix because the 3091 interactions occur only between the transcription factors (TFs) (152) and regulated genes (1146). This network is assumed as the true network to assess the performance of the inference algorithms. We would like to point out that this network respectively its interactions have been assembled in 2007 by [23]. That means, interactions added to RegulonDB or other databases after that date are not included. For the estimation of mutual information values, a B-spline smoothing and discretization method [44] has been used, with 10 bins and 3rd order B-splines, as in [23]. By using the same estimates as in [23] for the mutual information values, our results reveal differences due to methodological differences only, not effected by the usage of different MI estimators.

## Results and Discussion

We start our numerical analysis of C3NET by using simulated ensemble data. After that we investigate C3NET with expression data from *E. coli*.

### Simulated data

**Figure 2:**
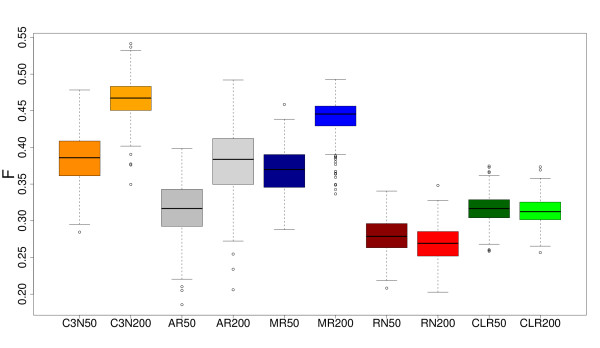
**Boxplots of F-scores for C3NET (orange), ARACNE (gray), MRNET (blue), RN (red) and CLR (green)**. Dark color (left boxplot) corresponds to sample size 50, light color (right boxplot) to sample size 200. A subnetwork of Yeast GRN is used for the simulations. Ensemble size is *N *= 300.

We compare the performance of C3NET with four of the most prominent inference algorithms, ARACNE [24], MRNET [25], RN [22] and CLR [23], that are widely used in the literature. Fig. 2 shows the boxplots of the resulting F-scores for two different sample sizes (*p *= {50, 200}) as indicated by the number behind the name of an inference algorithm. As underlying network structure a subnetwork of the TRN of yeast [34] is used. For both sample sizes one can see that C3NET provides better results than all four other inference algorithms, as indicated by the median value of the F-score. Also, with respect to other statistics, e.g., maximum, minimum or mean F-scores, C3NET provides better results than all other methods. A summary of these numerical results is provided in Table 1.

**Table 1:** Summary of F-scores (max, min, mean and median) for C3NET, ARACNE and MRNET obtained from our simulations.

		C3NET	ARACNE	MRNET
Yeast _200_	max	0.5478	0.4919	0.4927
	min	0.336	0.2058	0.336
	median	0.4628	0.3836	0.4455
	mean	0.4628	0.3795	0.4410

Yeast _50_	max	0.4782	0.3983	0.4585
	min	0.2844	0.1854	0.2879
	median	0.3859	0.3166	0.3698
	mean	0.3848	0.3161	0.3683

Ecoli	max	0.6046	0.4973	0.5608
	min	0.4131	0.1866	0.3512
	median	0.5308	0.3803	0.500
	mean	0.5269	0.3758	0.4948

The sample size is 1000 for *E. coli*, for Yeast we used 200 and 50 as indicated by the number in the corresponding rows.

**Figure 3:**
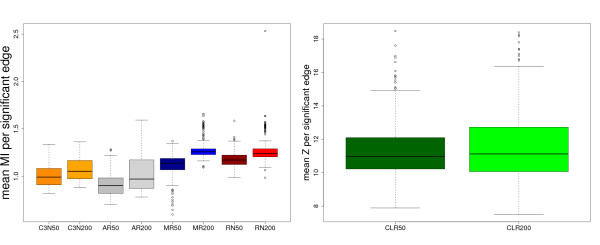
**Boxplots for the average mutual information values respectively z-scores per significant edge for C3NET (orange), ARACNE (gray), MRNET (blue), RN (red) and CLR (green)**. Dark color (left boxplot) corresponds to sample size 50, light color (right boxplot) to sample size 200. A subnetwork of Yeast GRN is used for the simulations. Ensemble size is *N *= 300.

The boxplots in Fig. 3 show the mean MI values per significant edge, respectively the mean z-score for CLR. Here, the notion *mean *indicates an averaging over all significant edges in a network. Edges are called *significant *if they are selected by the inference algorithm. The behavior of the resulting boxplots, for example with respect to the median values, but also other entities like the maxima, is a reflection of the intricate heterogeneity of the MI values that depend on structural elements of the network. We referred earlier in this paper to this phenomenon when motivating our approach. Here we just want to point out that high mean MI values per significant edge do not inevitably lead to high F-scores, because a significant edge can be a TP or a FP. Hence, the extremal selection mechanism employed by our algorithm seems to work in favor for avoiding FP edges despite the fact that their MI values may be quite high.

**Figure 4:**
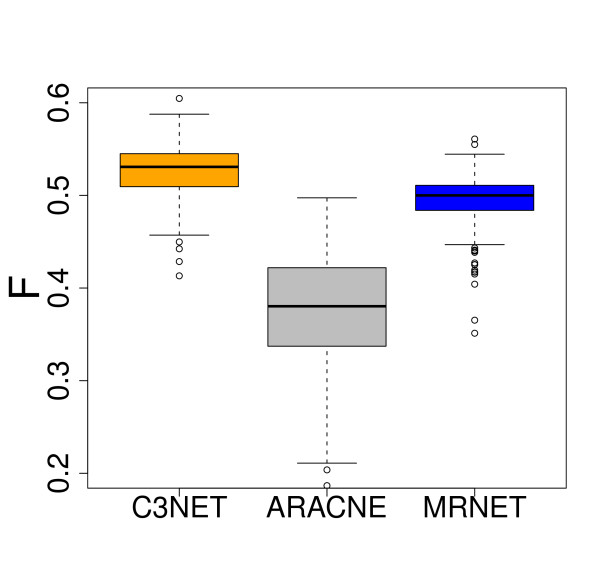
**Boxplots for the F-scores for C3NET (orange), ARACNE (gray) and MRNET (blue)**. A subnetwork of the TRN of *E. coli *is used for the simulations. Sample size is 1000 and ensemble size is *N *= 300.

In order to study the influence of the underlying network structure we repeat our analysis, this time, using a subnetwork of *E. coli *[32,33]. Again, we use an ensemble of size 300 resulting in 300 different data sets, each consisting of sample size 1000. The data were generated in the same way as for yeast. For our analysis we use the three best performing algorithms C3NET, ARACNE and MRNET, according to our analysis for yeast. The results of our simulations are shown in the boxplots in Fig. 4. Here we observe that despite the usage of a different network and different sample sizes, we essentially confirm our results obtained for yeast, indicating that C3NET provides the best results. This provides strong evidence that the results obtained for C3NET are robust with respect to a variability inevitably present in the data. Table 1 provides a summary of the obtained results for the subnetworks of Yeast and *E. coli*, for various sample sizes we used, giving the maximum, minimum, median and mean F-score values for the inference algorithms. One can see that C3NET gives in 11 out of 12 cases the best result and in the one remaining cases it is quite close to the best performing method.

**Figure 5:**
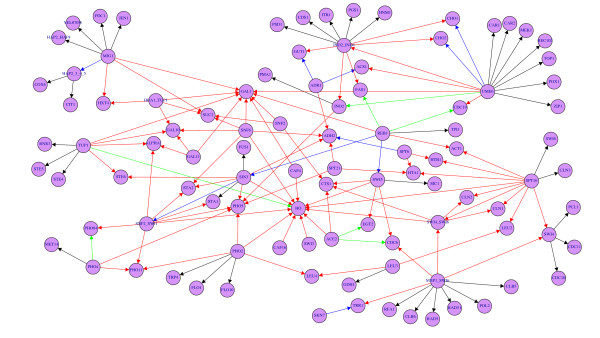
**Subnetwork of yeast consisting of 100 genes, sample size is 200**. Edge colors are obtained from simulations of 300 data sets. The color of each edge reflects its mean TPR. Specifically, for black edges, $1\geq \bar{TPR}>0.75$, for blue edges, $0.75\geq \bar{TPR}>0.5$, for green edges, $0.5\geq \bar{TPR}>0.25$, and for red edges, $0.25\geq \bar{TPR}\geq 0.0$.

**Figure 6:**
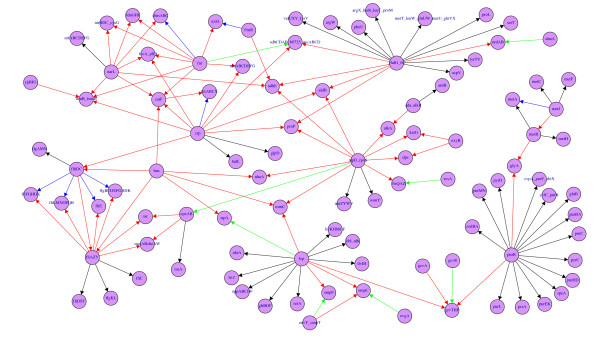
**Subnetwork of *E. coli *consisting of 100 genes, sample size is 1000**. Edge colors are obtained in a similar way as for yeast. Ensemble size is 300.

In Fig. 5 and 6 we show the true subnetwork of Yeast and *E. coli *used in our simulation study. The labels of the nodes correspond to gene names and the color of the edges visualizes the mean true positive rate $\left(\bar{TPR}\right)$ of the corresponding edges with the color code: black edges, $1\geq \bar{TPR}>0.75$, blue edges, $0.75\geq \bar{TPR}>0.5$, green edges, $0.5\geq \bar{TPR}>0.25$, and red edges, $0.25\geq \bar{TPR}\geq 0.0$. One can observe that C3NET infers all leaf edges correctly because the edges connecting to leaf nodes are black in both networks. A node is called a leaf node if it has only one incoming edge and no outgoing edges. The incoming edge is called leaf edge. From this observation one can hypothesize that C3NET is in general strong in inferring leaf edges. Further, from studying red edges it can be observed that colliders, a node that has two incoming edges, causes difficulties for the respective edges. One can see this, e.g., for the high-affinity glucose transporter HXT4 or glycoprotein PHO11 for yeast or for the proline/glycine betaine transporter proP or 3-methyl-adenine DNA glycosylase II alkA for *E. coli*. However, counter examples can be found as well, e.g., acetyl-coA synthetase isoform ACS1 for yeast which is connected by one red and one blue edge. Also, the collider homoserine O-transsuccinylase metA for *E. coli *can be inferred indicated by one black and one blue edge. Hence, for colliders the situation is much more involved than for leaf nodes, making a general prediction about the inferability of the respective edges difficult. For the hub-like nodes in yeast, UME6 (transcriptional regulator), INO2 (transcription activator) or MBP1 (transcription factor), the inferability of connected edges depends crucially on the type of the nodes. If the nodes are leaf nodes they can be inferred, if they are not they are more difficult to infer. Similar observations hold for *E. coli*. Hence, hubs do not appear to be easier to detect, however, due to the fact that they are per definition connected to many other nodes, there is a fair chance that one or more of these nodes may be a leaf node. For this reason, they are more likely to appear in the inferred network.

### Expression data from *E. coli*

Next, we apply C3NET to expression data from *E. coli*. We use the data set from [23] consisting of 524 microarrays. For this data set it has been shown that CLR provides better results than ARACNE and RN [23], by using a manually assembled reference network, $G_{2007}^{EC}$, considered as true regulatory network. For this reason, we compare in the following only C3NET with CLR.

Following a similar approach for CLR, as described in [23], we obtain a threshold value of 6.974 for the z-scores used by CLR. In summary, CLR predicts a total of 274 interactions from the 152 transcription factors to all other 1146 regulated genes. This results in TP = 169 (true positives), *FP *= 105 (false positives), *FN *= 2922 (false negatives) and a precision of 0.62.

For the significance test of the mutual information values we obtain a threshold value of 0.414. Application of C3NET results in a total of 99 interactions of which *TP *= 74 (true positives), *FP *= 25 (false positives), *FN *= 3017 (false negatives) giving a precision of 0.75. Comparison of the results for C3NET with the results for CLR shows that overall C3NET declares fewer edges as significant (99 for C3NET and 279 for CLR). Among these, the number of true positives is by 43% lower than for CLR. More importantly, C3NET reduces the number of false positives by 76% over CLR. Taken together, this explains the overall gain in precision we observe. Hence, the results for C3NET are more conservative, as expected, resulting in less significant edges. However, the quality of these edges, as measured either by the number of false positive edges or the precision, increases substantially over CLR. From a practical point of view this is desirable because a lower number of false positive edges means a lower number of false positive interactions, reducing the risk of negative results if tested experimentally in the laboratory.

**Figure 7:**
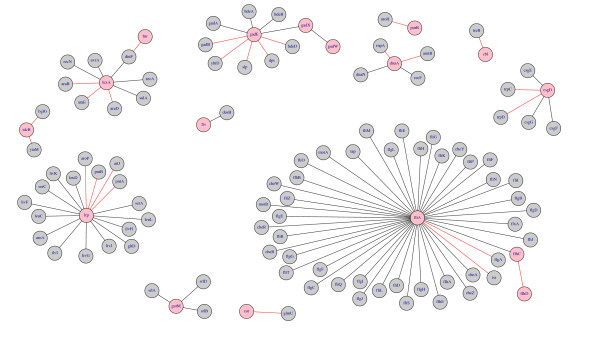
**Inferred *E. coli *network by C3NET**. Pink genes correspond to transcription factors and gray genes to regulated genes. Black edges indicate true positive results whereas red edges correspond to false positives.

Fig. 7 shows the inferred network of *E. coli *obtained by C3NET. In this figure black edges correspond to true positive edges and red edges correspond to false positive edges, as declared by using the reference network. Gray genes correspond to regulated genes and pink genes to regulating (transcription factors) genes.

In this network, the largest hub inferred by C3NET is fliA, a RNA polymerase. FliA is a minor sigma factor responsible for the initiation of transcription and involved in motility. The second largest hub in the inferred network is Lrp. The leucine-responsive protein (Lrp) is a transcription regulator widely distributed throughout archaea and eubacteria [45,46] and in *E. coli *involved in the regulation of the nitrogen metabolism [47]. C3NET inferred 17 interactions for Lrp, whereas 13 of them are true positives. Experimental results in [47] find that Lrp indirectly regulates the general aromatic amino acid transporter (aroP) via TyrR. That means the causal chain of regulation is Lrp → TyrR → aroP. Hence, a direct connection between Lrp and aroP, as inferred by C3NET, is the classic case of a false positive where the algorithm failes to discriminate between causation and association, because apparently aroP is indirectly regulated by Lrp. Further, C3NET infers 8 interactions for lexA, a DNA-binding transcriptional repressor of the SOS regulon and related to DNA repair and cell division, whereas 5 of them are true positives; and 5 interactions for csgD of which 3 are true positives.

Table 2 lists all false positive results of C3NET, shown as red edges in Fig. 7. Because the reference network, $G_{2007}^{EC}$, was assembled in 2007 we repeated a literature search taking into account all recent publications in order to clarify the actual state of the interactions listed in table 2. From our literature search we find five interactions, listed at the top of table 2, for which supporting information can be provided that they actually correspond to real biological interactions.

**Table 2:** Interactions predicted by C3NET, shown as red edges in Fig. 7, declared as false positives according to the reference network $G_{2007}^{EC}$.

	regulator gene	regulated gene	literature
confirmed	gadE	gadB	[49]
	gadE	hdeD	[49]
	gadE	yhiD	[50]
	lrp	pntA	[51]
	fliA	tsr	[52]
	flhD	flhC	[53] (protein complex)

predicted interactions	dnaA	amiB	
	dnaA	rnpA	
	fliA	flgA	
	lrp	artJ	
	lrp	aroP	indirect interaction via TyrR [47]
	lexA	araB	
	lexA	araD	
	lexA	araE	
	lrp	pntB	
	tdcR	yiaM	
	tdcR	bglG	
	csgD	trpD	
	csgD	trpC	
	zur	glmU	
	purR	aroH	
	fnr	dinF	
	cbl	treB	
	gadE	slp	
	gadE	dps	

Among these 25 interactions 6 receive support from the literature to be in fact true positives.

GadE is an essential transcriptional activator of the glutamate decarboxylase (GAD) system which is reported to be the most efficient acid resistance (AR) mechanism in *E. coli *[48]. For GadE, C3NET predicted three interactions we could verify, one between gadE and gadB, one between gadE and hdeD and the third between gadE and yhiD. For all three interactions we find experimental support in [49] and [50]. From ChIP-chip experiments [51] it was found that lrp regulates pntA. Finally, by studying flagellar and chemotaxis [52] find that fliA transcribes tsr.

In addition to these five transcription regulations we find support for a different type of interaction, namely a protein-protein binding. In [53] it is reported that the flhD operon encodes two genes, flhD and flhC, which code for two proteins, FlhD and FlhC, forming a protein complex. Further, they showed that the FlhD/FlhC complex had a DNA-binding activity and binds to the upstream regions of fliA, flhB, and fliL operons (class II), which are under direct control of the flhD operon [53]. It is worth mentioning that by using expression data providing information about the concentration of mRNAs, no method can guarantee what type of interaction is actually inferred. Because the expression data itself do not provide a direct evidence for any type of molecular interaction, such as transcription regulation, protein-protein interaction or methylation, instead, these data provide dynamical information that are a consequence of the aforementioned interactions. For this reason, it should not be surprising to detect, in addition to the regulation of transcription, also further interaction types, as the last interaction pair demonstrates. Among the remaining predicted interactions listed in table 2, there are a couple of further candidates that could potentially be true positives. However, additional experimental evidence is needed to faithfully demonstrate this. For this reason, in this table we declare those remaining interactions as *predicted interactions *because one can also not provide evidence that these interactions do *not *exist in *E. coli*.

Taking these newly confirmed interactions into account, the precision of C3NET increases to 0.81. Finally, we want to report that [54] find several putative interactions among lrp and gadE and between gadE and fliA. Due to the fact that lrp and fliA form hubs in our network and we know from our simulation studies that connections between such hubs are difficult to infer with C3NET, these putative interactions may be present in the regulatory network of *E. coli *as well.

## Conclusions

In this paper we introduced a novel unsupervised GRNI method, called C3NET, in order to infer causal regulatory networks. We investigated the performance of C3NET by conducting in-depth simulations using 900 synthetic data sets in combination with two different subnetworks from yeast and *E. coli*, and also large-scale expression data from *E. coli*. From these studies and the comparison with several well-known inference methods frequently used, namely, ARACNE, CLR, MRNET and RN, we find that C3NET provides consistently better results. For example, for the expression data from *E. coli*, C3NET gives a precision of 0.81 which is an increase of about 31% compared to the precision obtained for CLR, which in turn was demonstrated to perform better than ARACNE and RN for this data set [23].

The conservative approach of C3NET, allowing each gene to contribute (add) at most one edge to the inferred network, appears to exploit the estimates of mutual information values significantly better than previous methods. The simplicity of our approach demonstrates that it is not always favorable to increase the complexity of an inference procedure in order to increase its performance. More important is a concise design that takes the nature and constraints of the underlying problem into account. Also, the investigation of an inference method using simulated ensemble data is strongly advised to obtain a clear assessment of such a method, because the results obtained for individual data sets may be atypical. In contrast, ensemble data uncover relentlessly the entire spectra of behavior an inference method can exhibit. Hence, an important result from our study is the insight that a neatly structured algorithm can perform better than other methods that are more complex. This is not only favorable because it allows a better understanding of the inference procedure itself but usually leads to more robust results, especially when the sample size is small.

Although, our method has been invented for the inference of gene regulatory networks applied to expression data, it may find application in other fields as well that aim at inferring causal relations among covariates, because the requirements for the data are moderate. For example, C3NET could find its application for the inference of brain connectivity networks [55].

## Authors' contributions

GA and FES designed the method, performed the analysis and interpreted the results. FES conceived and coordinated the study. GA and FES wrote the manuscript. All authors read and approved the final manuscript.

## Appendix

For our numerical simulations we used R [56], SynTRen [35] and MINET [42] and for the visualization of the networks the igraph package [57].
